# Time trend of prevalence of self-reported cataract and its association with prolonged sitting in Taiwan from 2001 and 2013

**DOI:** 10.1186/1471-2415-14-128

**Published:** 2014-11-05

**Authors:** Ya-Hui Shih, Hsing-Yi Chang, Ming-Ing Lu, Baai-Shyun Hurng

**Affiliations:** Division of Preventive Medicine and Health Services Research, Institute of Population Health Sciences, National Health Research Institutes, 35 Keyan Road, Zhunan Town, Miaoli County, 350 Taiwan; Health Promotion Administration, Ministry of Health and Welfare, Taichung, Taiwan

**Keywords:** Prevalence of cataract, Prolonged sitting, National Health Interview Survey, Taiwan

## Abstract

**Background:**

Prolong sitting has been found associated with metabolic disorders. Little is known about the self-reported cataract status in general population of Taiwan, not to mention its relation to prolong sitting. We aimed to examine the prevalence of cataract between 2001 and 2013 in Taiwan and to the association between prolonged sitting and cataract.

**Methods:**

We used three data sets with those aged 40 years and older from the National Health Interview Survey (NHIS) from 2001 (n = 8334), 2009 (n = 11207), and 2013 (n = 10940). Subsequent statistical analyses involved chi-square test, *t* test, and logistic regression modeling. SUDAAN was used to account for sampling scheme.

**Results:**

The prevalence of cataract ranged from 10.7% in 2001, 13.13% in 2009, to 11.84% in 2013. Participants who sat for more than 7 hours per day had a significantly higher risk of cataract (OR = 1.20, CI = (1.04-1.39)) compared with those who sat for fewer than 3 hours per day after controlling for age and other risk factors like being older or female, lower education level, not being currently employed, living in a highly urbanized area, having hypertension, diabetes, myopia, and being an former smoker (compared to a never smoker).

**Conclusion:**

Increased daily sitting time was associated with cataract, especially for people who sat more than 7 hours per day.

## Background

According to 2010 World Health Organization (WHO) data, there are 39 million persons who are blind worldwide, with the three leading causes of blindness being cataract (51%), glaucoma (8%), and age-related macular degeneration (5%) [1]. In addition to being an important cause of blindness, cataract also leads to high health-care costs. According to US research published in 2004, cataracts represent 19.21% of total expenditure on eye health care in those aged 40 years and older and an even greater proportion of expenditure (68.6%) in those aged 65 years and older [2]. The prevalence of cataract has a very strong positive relationship with age [3]. Taiwan officially became an ageing society in 1993, and in 2012 the proportion of the population aged 65 years and older reached 11.2%. This proportion is expected to reach 14% in 2018, making Taiwan an aged society, and then surpass 20% in 2025, making Taiwan a super-aged society. Therefore, diseases that have a strong relationship with older age, such as cataract, require increased attention and disease control efforts.

A cataract is a clouding of the lens inside the eye which leads to a decrease in vision. It is mostly due to biological aging [4]. Other factors including trauma, radiation [5], skin diseases, smoking [6] and use of corticosteroids [7] are known to cause cataract. In addition, factors include hypertension [8], diabetes [9, 10], and myopia [11] are also found to be associated with cataract. Previous research found that individuals living in rural areas were more likely to have certain types of cataracts [11]; but, another study reported that urban dwellers were more likely to have cataracts [12].

Nowadays, people spend a lot of time staring at computer screen, watching TV, or using smart phones or tablets. These electronic devices might produce photo-toxicity [13]. Long time exposure to photo-toxicity could damage lens protein, thus induce early onset of cataract [14]. The length of time spent sitting could possibly have an impact on eye health. A small number of studies have found an association between a sedentary lifestyle and visual impairment in older persons [15, 16] and age-related macular degeneration [17]. Sedentary behavior is also associated with visual function in people with diabetes [18, 19]. Prolonged sitting and lack of physical activity are both distinct risk factors for cataract, and even those who frequently exercise can spend prolonged time sitting in front of a TV [20–22]. It is very likely to induce cataract.

There has been less research conducted in Taiwan about the prevalence of cataract and its associated factors. Most of these researches have focused on small populations older than 50 years in specific localities [23, 24]. Little is known about the cataract status in general population of Taiwan, not to mention its relation to prolong sitting. In this study, we used the National Health Interview survey to examine the time trend of cataract and its association with prolong sitting time.

## Methods

Data for this study were from the 2001, 2009 and 2013 National Health Interview Surveys (NHIS). These surveys used a multistage stratified systematic sampling design. In 2001, it stratified the whole Taiwan area into seven strata according to the degree of urbanization, geographic location, and administrative boundaries sampled. Individuals were sampled with probability proportional to size [25]. The target population for the survey was individuals whose households were registered in any one of the 23 counties or cities in Taiwan at the end of the year prior to the survey. In the 2001 survey, households were the basic sampling unit, whereas in the 2009 and 2013 survey, individuals were the basic sampling unit. Written informed consent for participation in the study was obtained from participants (≥19 years), where participants were children (<19 years), a parent or guardian. Comparison was carried out between the sample and the target population, and none of the chi-square tests demonstrated a statistically significant difference.

In the present study we used the Taiwanese sample weights provided by the NHIS working group to carry out weighting for 2009 and 2013. The response rates for the 2001, 2009 and 2013 surveys were 93.8% [25], 83.96% and 75.2%, respectively. Further details regarding questionnaire content and design and sampling design are provided on the NHIS website (http://nhis.nhri.org.tw/) [25]. On the same website, researchers and government workers can apply for the survey data. The NHIS was designed to be carried out every 4 years, but the 2005 survey did not include a measure of time spent sitting for participants those aged 65 years and over. Therefore, we were only able to use data from the 2001, 2009 and 2013 surveys.

Participants aged 40 years and older were included in the current analysis. Outcome variable was the self-reported current status of cataract (told by medical professionals) in one or two eyes. Explanatory variables included age, sex, education level, marital status, employment status, monthly household income, degree of urbanization, health status (hypertension, diabetes, and myopia), smoking status, and daily sitting time. Disease of hypertension, diabetes, and myopia were self-reported and told by a medical professional. This set of questions has been used since 2001. We have validated the question on diabetes in 2002, when we had measurements and blood samples from half of the original sample aged 15 and above. Among the 2002 survey, 86% of females and 77% of males were aware of their disease status [26]. That indicated the agreement was reasonable. Age was divided into three groups: 40–54 years, 55–64 years, and 65 years and above. Education level was categorized as none (without any education), junior high school and below, senior high school, college or Bachelor’s degree, and Master’s degree and above. Participants who had studied at an online university or professional college were categorized as having a college or Bachelor’s degree. Marital status was categorized as never married, married, and other (including living together, separated, divorced, and widowed). Employment status referred to the response to the question “Are you currently working?” Participants who had previously worked were categorized as “no” if they were not currently working. Monthly income was categorized as < NT$30,000, NT$30,000 to < NT$70,000, ≥NT$70,000 (1NT ≈ 0.033USD), and “not stated” (including unwilling to report, unknown, and unclear). Degrees of urbanization were categorized as high urbanization, moderate urbanization, developing towns, general towns, and other towns (including aged towns, agricultural towns, and villages). Smoking status was categorized as never, former smokers, or current. Daily sitting time was assessed by the question “How many hours do you spend sitting on an average day, including time spent at work, at school, driving, reading books, reading the newspapers and using the computer?” Responses were categorized as <3 hours, 3–4 hours, 5–6 hours, and 7 hours or more, which were approximate quartiles of the data. These cut-points were also used in other studies [27–29]. We performed imputations for missing value of daily sitting time (1.7%) by using age, gender, education, employed status and marital status as imputation data.

Statistical analyses were carried out using SAS 9.3 software and the SUrvey DAta ANalysis (SUDAAN) to account for sampling schemes. Descriptive statistics were used to examine the distribution of basic characteristics. Trend test was used to assess time trend between socio-demographical factors, health status, and other variables and three surveys. Chi-square test and *t* test were applied to compare risk factors between cataract and no cataract. Logistic regression was used to determine the association between daily sitting time and the presence of cataract controlling for demographic variables and other risk factors.

## Results

In the 2001, 2009 and 2013 NHIS data, there were 8,334, 11,207 and 10,940 respondents aged 40 years and older respectively (Table 1). The prevalence of cataract ranged from 10.7% in 2001 to 11.84% in 2013. Daily sitting time and the mean age was higher in 2013 than in 2001 (p < 0.001). Around 25% of people sat more than 7 hours per day in 2001 and 2009, and increased to more than 30% in 2013. The proportion of elderly (≥65 years old) reached 23.19% in 2013, where it was 24.68% in 2001. People in 2013 had higher education levels than those in 2001. A higher proportion of people were married in 2001 than in 2013. People living in highly urbanized areas were higher in 2013 than in 2001 but those livings in other towns areas were higher in 2001 than in 2013. More people in 2013 had hypertension, diabetes, and myopia than those in 2001. Table 2 compared the characteristics of those with cataract to those without in all surveys. Almost all variables like age, sex, education level, marital status, employment status, monthly income, health status, and smoking were associated with cataract, except sitting time in 2001 and 2009 and degrees of urbanization in 2001.

**Table 1:** **Characteristics of the study sample aged 40 years and older in three surveys**

Variable	Year 2001		Year 2009		Year 2013		P value*
	n	(%)	N	(%)	N	(%)	
**Total**	8334		11207		10940		
**Mean sitting time (SD)**	5.17 ± 0.09		5.38 ± 0.06		5.69 ± 0.05		<0.001
**Sitting time (grouped), hours**							<0.001
<3	1892	(22.70)	2100	(18.74)	1624	(14.84)	
3–4	2421	(29.05)	3198	(28.54)	2993	(27.36)	
5–6	1803	(21.63)	2820	(25.17)	2852	(26.07)	
≥7	2218	(26.61)	3088	(24.55)	3471	(31.73)	
**Mean age (SD)**	56.11 ± 0.26		56.31 ± 0.16		57.04 ± 0.17		<0.001
**Age (grouped), years**							0.055
40–54	4586	(55.03)	6104	(54.46)	5507	(50.34)	
55–64	1691	(20.29)	2474	(22.08)	2896	(26.47)	
≥ 65	2057	(24.68)	2629	(23.46)	2538	(23.19)	
**Sex**							0.697
Male	4086	(49.39)	5535	(49.39)	5336	(48.77)	
Female	4248	(50.97)	5671	(50.61)	5605	(50.23)	
**Education level†**							0.006
None	1458	(17.49)	1018	(9.08)	839	(7.67)	
Junior high school and below	4127	(49.52)	4883	(43.57)	4265	(38.98)	
Senior high school	1572	(18.86)	2995	(26.72)	3051	(27.89)	
College or Bachelor’s degree	1090	(13.08)	1993	(17.78)	2267	(20.73)	
Master’s degree and above	87	(1.04)	319	(2.84)	518	(4.73)	
**Marital status** **‡**							<0.001
Never married	262	(3.14)	596	(5.31)	787	(7.19)	
Married	6737	(80.84)	8566	(76.43)	8129	(74.30)	
Other	1335	(16.02)	2045	(18.25)	2025	(18.51)	
**Employed status**	4084	(49.00)	6318	(56.38)	6373	(58.25)	<0.001
**Monthly household income ($NT)**							<0.001
<30,000	2101	(25.21)	2849	(25.42)	2266	(20.71)	
≥ 30,000 to <70,000	3444	(41.32)	3775	(33.68)	3751	(34.28)	
≥70,000	2687	(32.24)	2650	(23.65)	3170	(28.98)	
Not stated	102	(1.22)	1932	(17.24)	1753	(16.03)	
**Degrees of urbanization§**							<0.001
High urbanization	1769	(21.23)	2530	(22.58)	2948	(26.95)	
Moderate urbanization	2249	(26.99)	2558	(22.82)	5369	(49.07)	
Developing towns	1827	(21.92)	3598	(32.11)	1353	(12.37)	
General towns	1226	(14.71)	1802	(16.08)	884	(8.08)	
Other towns	1263	(15.15)	719	(6.41)	387	(3.53)	
**Health status**							
Cataract	882	(10.70)	1471	(13.13)	1296	(11.84)	0.122
Hypertension	1825	(21.90)	2934	(26.18)	3141	(28.71)	<0.001
Diabetes	759	(9.11)	1173	(10.47)	1302	(11.90)	<0.001
Myopia	1641	(19.69)	3384	(30.19)	3839	(35.09)	<0.001
**Smoking status**							<0.001
Never smokers	5872	(70.46)	7077	(63.15)	7157	(65.42)	
Former smokers	477	(5.72)	1695	(15.13)	1569	(14.34)	
Current smokers	1985	(23.82)	2434	(21.72)	2214	(20.24)	

*P value based on test of trend.

†Education level: College and above includes online universities and online professional colleges.

‡Marital status: other includes living together, separated, divorced, and widowed.

§Degrees of urbanization: other towns include aged towns, agricultural towns, and villages.

**Table 2:** **Factors associated with cataract in those aged 40 years and older in three surveys**

Variable	Year 2001		P value*	Year 2009		P value*	Year 2013		P value*
	N	(%)		N	(%)		N	(%)	
**Total**	892	(10.70)		1471	(13.13)		1296	(11.84)	
**Mean sitting time (SD)**	5.44 ± 0.14		0.040	5.39 ± 0.12		0.725	5.66 ± 0.12		0.665
**Sitting time (grouped), hours**			0.088			0.487			0.037
<3	195	(10.31)		303	(14.44)		218	(13.40)	
3–4	236	(9.75)		414	(12.94)		313	(10.45)	
5–6	187	(10.37)		366	(12.97)		369	(12.94)	
≥7	274	(12.35)		388	(12.57)		396	(11.42)	
**Mean age (SD)**	70.12 ± 0.33		<0.001	71.88 ± 0.32		<0.001	70.87 ± 0.36		<0.001
**Age (grouped), years**			<0.001			<0.001			<0.001
40–54	61	(1.33)		80	(1.31)		71	(1.30)	
55–64	179	(10.59)		276	(11.15)		297	(10.27)	
≥ 65	652	(31.70)		1115	(42.42)		927	(36.53)	
**Sex**			<0.001			<0.001			<0.001
Male	364	(8.91)		610	(11.02)		531	(9.95)	
Female	528	(12.43)		861	(15.18)		765	(13.64)	
**Education level†**			<0.001			<0.001			<0.001
None	349	(23.94)		398	(39.08)		301	(35.92)	
Junior high school and below	400	(9.69)		773	(15.83)		614	(14.40)	
Senior high school	81	(5.15)		152	(5.09)		182	(5.96)	
College or Bachelor’s degree	55	(5.05)		128	(6.26)		157	(6.94)	
Master’s degree and above	7	(8.05)		23	(7.23)		41	(7.90)	
**Marital status‡**			<0.001			<0.001			<0.001
Never married	11	(4.20)		28	(4.63)		32	(4.03)	
Married	592	(8.79)		921	(10.75)		861	(10.59)	
Other	289	(21.65)		523	(25.55)		403	(19.92)	
**Employment status**			<0.001			<0.001			<0.001
Employed	125	(3.06)		230	(3.64)		283	(4.44)	
Not employed	767	(18.05)		1241	(25.38)		1013	(22.18)	
**Monthly household income ($NT)**			<0.001			<0.001			<0.001
<30,000	359	(17.09)		562	(19.72)		395	(17.42)	
≥ 30,000 to <70,000	304	(8.83)		317	(8.39)		360	(9.60)	
≥70,000	219	(8.15)		163	(6.16)		247	(7.78)	
Not stated	10	(9.80)		429	(22.21)		294	(16.79)	
**Degrees of urbanization§**			0.324			<0.001			0.002
High urbanization	188	(10.63)		388	(15.34)		396	(13.42)	
Moderate urbanization	235	(10.45)		346	(13.52)		560	(10.43)	
Developing towns	170	(9.30)		363	(10.10)		155	(11.43)	
General towns	127	(10.36)		268	(14.88)		127	(14.35)	
Other towns	172	(13.62)		105	(14.64)		59	(15.20)	
**Health status**									
**Hypertension**			<0.001			<0.001			<0.001
Yes	357	(19.56)		766	(26.12)		724	(23.4)	
No	535	(8.22)		705	(8.52)		572	(7.33)	
**Diabetes**			<0.001			<0.001			<0.001
Yes	187	(24.64)		345	(29.38)		328	(25.22)	
No	705	(9.31)		1126	(11.22)		967	(10.04)	
**Myopia**			<0.001			<0.001			<0.001
Yes	84	(5.12)		210	(6.20)		251	(6.54)	
No	808	(12.07)		1261	(16.12)		1045	(14.71)	
**Smoking status**			<0.001			<0.001			<0.001
Never smokers	673	(11.46)		1019	(14.40)		979	(13.67)	
Former smokers	83	(17.40)		286	(16.88)		211	(13.43)	
Current smokers	136	(6.85)		166	(6.81)		106	(4.80)	

*P value based on chi-square test for categorical variables and t-test for continuous variables (cataract versus no cataract).

†Education level: College and above includes online universities and online professional colleges.

‡Marital status: other includes living together, separated, divorced, and widowed.

§Degrees of urbanization: other towns include aged towns, agricultural towns, and villages.

Table 3 shows logistic regression results for potential factors associated with the presence of cataract. After controlling for all potential associated factors, the odds ratio for the presence of cataract was 1.20 (p = 0.016) for those sitting for 7 or more hours per day compared to those sitting for less than 3 hours per day. The probability of having cataract in 2009 was significantly higher compared with 2001 (OR = 1.42, p < 0.001). The probability of having cataract increased with increasing age (p < 0.001). Women had a greater risk of cataract than men (OR = 1.33, p < 0.001). Those with a junior high school and below were less likely to have cataract than those without education (OR = 0.85, p = 0.020). Similar pattern was found in those with a senior high school (OR = 0.71, p = 0.001) and college or Bachelor’s degree (OR = 0.77, p = 0.028). Participants who were currently employed were less likely to have cataract (OR = 0.61, p < 0.001). Participants living in highly urbanized areas were more likely to have cataract than those living in other levels of urbanization (p < 0.01). Participants with hypertension (OR = 1.39, p < 0.001), diabetes (OR = 1.58, p < 0.001), or myopia (OR = 1.26, p = 0.008) were more likely to have cataract. In addition, those who were former smokers were more likely to have cataract (OR = 1.20, p = 0.046) and those who were current smokers were less likely to have cataract than those who were never smokers (OR = 0.83, p = 0.045).

**Table 3:** **Logistic regression analysis of factors associated with cataract in Taiwanese persons aged 40 years and older**

Variable	Category	OR	95% CI	P value
**Survey year**				
	2001	**1.00**		
	2009	1.42	(1.23 - 1.63)	<0.001
	2013	1.07	(0.93 - 1.23)	0.335
**Sitting time (grouped), hours**				
	<3	**1.00**		
	3–4	1.09	(0.95 - 1.25)	0.226
	5–6	1.02	(0.88 - 1.18)	0.783
	≥7	1.20	(1.04 - 1.39)	0.016
**Age (group), years**				
	40–54	0.04	(0.03 - 0.05)	<0.001
	55–64	0.25	(0.22 - 0.29)	<0.001
	≥ 65	**1.00**		
**Sex**				
	Male	**1.00**		
	Female	1.33	(1.16 - 1.53)	<0.001
**Education level†**				
	None	**1.00**		
	Junior high school and below	0.85	(0.74 - 0.97)	0.020
	Senior high school	0.71	(0.59 - 0.87)	0.001
	College or Bachelor’s degree	0.77	(0.61 - 0.97)	0.028
	Master’s degree and above	1.03	(0.64 - 1.66)	0.899
**Marital status‡**				
	Never married	0.83	(0.58 - 1.18)	0.282
	Married	**1.00**		
	Other	1.09	(0.98 - 1.23)	0.121
**Employed status**		0.61	(0.53 - 0.70)	<0.001
**Monthly household income ($NT)**				
	<30,000	**1.00**		
	≥ 30,000 to <70,000	0.89	(0.78 - 1.02)	0.098
	≥70,000	0.87	(0.74 - 1.03)	0.102
	Not stated	0.82	(0.69 - 0.96)	0.018
**Degrees of urbanization§**				
	High urbanization	**1.00**		
	Moderate urbanization	0.78	(0.68 - 0.90)	0.001
	Developing towns	0.64	(0.54 - 0.76)	<0.001
	General towns	0.71	(0.59 - 0.85)	<0.001
	Other towns	0.68	(0.53 - 0.87)	0.003
**Health status**				
	Hypertension	1.39	(1.25 - 1.54)	<0.001
	Diabetes	1.58	(1.38 - 1.80)	<0.001
	Myopia	1.26	(1.06 - 1.48)	0.008
**Smoking status**				
	Never smokers	**1.00**		
	Former smokers	1.20	(1.00 - 1.56)	0.046
	Current smokers	0.83	(0.70 - 1.00)	0.045

†Education level: College and above includes online universities and online professional colleges.

‡Marital status: other includes living together, separated, divorced, and widowed.

§Degrees of urbanization: other towns include aged towns, agricultural towns, and villages.

## Discussion

In this study we used data from three large-scale, nationally representative samples collected 12 years apart and examined daily sitting time in an analysis of risk factors for cataract. Results showed that risk of cataract significantly (p < 0.05) increased with longer daily sitting time controlling for other risk factors. Our results confirmed our two study hypotheses that prolonged sitting would increase the risk of cataract.

The total number of persons with cataract increased 31.27% in 2013 compared to 2001. The overall prevalence increased by 2.43% in 2009 and 1.14% in 2013, which demonstrates the growth of cataract disease in Taiwan during this period. However, this growth may be due to several other factors. People in 2013 had higher mean age, higher education levels and there were more people living in high urbanization area than those in 2001. This change of characteristics of study sample may affect self-report of cataracts in recent surveys.

The prevalence of cataract in Taiwan is low compared with other countries. If we take the prevalence at age 40 years and older, for example, the prevalence in the United States is 22% [30]; in Asian regions such as Tibet [31], South Korea [32], India [33], Malaysia [34], Singapore [3], Sri Lanka [35], and Myanmar [36], the prevalence is at least 20% or more, with the highest over 50% [3]. The prevalence rates found in the present study are also lower than those found in previous surveys in the Peitou and Shihpai regions of Taiwan (prevalence rates of more than 50% were found) [23, 24]. These observed differences could be due to the method of ascertainment. We used a self-reported disease status. We emphasized “currently been told by a medical professional” during the survey interview in order to obtain diagnosed cases of cataract. However, this did not include individuals who were at early stage of cataract and had not been examined by a doctor. Omitting these persons could result in underestimate the prevalence.

Nevertheless, we observed the existence of self-reported cataract was associated with pro-long sitting time. This is possibly related to screen time. Nowadays, people sit for long time are most likely exposed to higher-luminance displays, which emanate short wavelength blue-violet light or ultraviolet [13]. This light would have photochemical reaction with lenses and produces reactive oxygen species (ROS), which induces oxidative stress to the protein of lenses and become oxidative damage. That is oxidative stress-induced cataract [14]. However, a 2003 study [37] investigated the cumulative incidence of cataract over 10 years and found no statistically significant association between prolonged sitting and cataract in either eye or any type of cataract. Limited research has been conducted on prolonged sitting and the development of cataract. Some studies have found that a sedentary lifestyle is associated with reduced visual function in individuals with diabetes [18, 19]. In addition, older persons with a sedentary lifestyle are more likely to have visual impairment [15] or age-related macular degeneration [17]. We controlled the age and diabetes, prolong sitting time was still associated with cataract significantly.

In addition to the previously mentioned factors, being female and having low socioeconomic status (including low education, unemployment, and low monthly household income), hypertension, diabetes, and myopia were also associated with an increased risk of cataract in our study. These findings are similar to those of previous studies [8, 11, 30, 35, 38].

In terms of smoking, we found that past smokers had an increased likelihood of cataract and current smokers had a decreased likelihood of cataract. It is possible that past smokers had a strong addiction or have quit smoking due to illness. In contrast, current smokers could be maintaining a low level smoking habit (such as one or two cigarettes now and then) or may have only recently started smoking. Therefore, further analysis by quantity of tobacco consumed is needed for a clearer comparison. We found that the risk of cataract was higher among residents in highly urbanized areas, which is similar to findings reported by Xu et al. [12].

Our study has several limitations. First, we used the self-reported current status of cataract told by a medical professional. This may underestimate the prevalence of cataract in the population, since those at early stage might not be diagnosed. Second, we didn’t record the type of cataract (nuclear, cortical or posterior subcapsular cataract (PSC)). It was difficult to identify association between risk factors and specific pattern of cataract. Third, we did not measure the time spent watching television, using the computer, or reading, so we were unable to examine whether the association between sitting time and cataract depends on the activity involved. Finally, several variables shown to be associated with cataract in international research were not included: UV radiation, type of occupation (outdoors or office-based), and medications. Living area was used as a proxy for the sunlight exposure, the relationship between prolong sitting with cataract was still apparent after control for living area.

## Conclusion

Prolonged sitting is a risk factor for cataract disease, particularly in those who sit for more than 7 hours per day. Attention should be given to the activities while sitting.

### Ethics approval

This study was approved by the Institutional Review Board of the National Health Research Institutes.
